# Nanoscale origins of creep in calcium silicate hydrates

**DOI:** 10.1038/s41467-018-04174-z

**Published:** 2018-05-03

**Authors:** A. Morshedifard, S. Masoumi, M. J. Abdolhosseini Qomi

**Affiliations:** 1 Advanced Infrastructure Materials for Sustainability Laboratory (AIMS Lab), Department of Civil and Environmental Engineering, Henry Samueli School of Engineering, E4130 Engineering Gateway, University of California, Irvine, Irvine, CA 92697-2175 USA; 2 Centre for Infrastructure Engineering and Safety (CIES), School of Civil and Environmental Engineering, UNSW Australia, UNSW Sydney, Sydney, NSW 2052 Australia

## Abstract

The time-dependent response of structural materials dominates our aging infrastructure’s life expectancy and has important resilience implications. For calcium-silicate-hydrates, the glue of cement, nanoscale mechanisms underlying time-dependent phenomena are complex and remain poorly understood. This complexity originates in part from the inherent difficulty in studying nanoscale longtime phenomena in atomistic simulations. Herein, we propose a three-staged incremental stress-marching technique to overcome such limitations. The first stage unravels a stretched exponential relaxation, which is ubiquitous in glassy systems. When fully relaxed, the material behaves viscoelastically upon further loading, which is described by the standard solid model. By progressively increasing the interlayer water, the time-dependent response of calcium-silicate-hydrates exhibits a transition from viscoelastic to logarithmic creep. These findings bridge the gap between atomistic simulations and nanomechanical experimental measurements and pave the way for the design of reduced aging construction materials and other disordered systems such as metallic and oxide glasses.

## Abstract (web summary)

The nanoscale mechanisms behind the creep of calcium-silicate-hydrates remain difficult to model over long periods of time. Here, the authors use a three-staged incremental stress-marching technique to tie atomistic simulations and nanomechanical experimental measurements together.

## Introduction

Like most engineering materials, concrete exhibits a time-dependent response, e.g. creep, when subjected to sustained external or internal load. This type of deformation not only impacts the life expectancy of our aging concrete infrastructure and the associated environmental footprint incurred for its rehabilitation, but also has important resilience implications. Although dislocation^1^ and shear transformation zones^2,3^ are at the heart of creep in crystalline materials and metallic glasses, the nanoscale mechanisms underlying time-dependent deformation in cementitious materials are complex and still the subject of intensive fundamental research^4–7^. For calcium-silicate-hydrates (C-S-H), the main binding phase in cementitious materials, this complexity can in parts be attributed to the presence of nanoconfined water, the material’s layered structure at the nanoscale^8–10^, its globular texture at the mesoscale^11^ and its multiscale porous structure^12,13^. These complexities further hinder the inherent difficulty in studying nanoscale longtime phenomena that arise from 18 orders of magnitude difference between characteristic time scales in atomic vibrations and creep deformation.

The majority of the literature focuses on continuum^14,15^, micromechanics^16^, and coupled microstructure-continuum models^17^ to predict creep in cementitious materials. Currently, a few phenomenological models are available that are calibrated with the goal of predicting a comprehensive database of experimental results^18,19^. These phenomenological models are critical in predicting long-term deformation of concrete structures, yet their link to fundamental materials physics remains to be understood. Also, the measurement of creep deformation in cementitious materials is further complicated owing to coexistence of long-term hydration process^20^ and the accompanying shrinkage deformation. The portion of creep deformation that is not owing to the hydration process, i.e., nonaging creep, constitutes a significant part of concrete’s overall time-dependent deformation and is the subject of this study. In an effort to address nonaging creep in C-S-H, microprestress solidification theory proposes the existence of frozen eigenstresses^4^ that relax over time following a power/logarithmic *ε*−*t* relation. Yet, the origins of C-S-H’s creep and its link to chemical composition and molecular structure of C-S-H cannot be explained by such phenomenological models and remain vastly unexplored. This calls for a molecular-level investigation of mechanisms behind C-S-H time-dependent phenomena.

Molecular dynamics (MD) simulation is the standard approach to explore atomic level processes that occur at the nanoscale. The first challenge in modeling cementitious materials is constructing an initial atomic structure for simulation purposes. Many studies have concluded that a structurally imperfect Tobermorite is a satisfactory model for C-S-H ^9,21,22^, which is the strategy adopted in the current paper. The next challenge is deriving accurate interatomic interactions such that reasonable agreement with a wide range of experimental observations is obtained from simulations. Several empirical force fields have been applied or developed for modeling C-S-H such as ClayFF^23^, CSH-FF^24^, CementFF^25^, and ReaxFF^26^. For details, the interested reader is referred to a recent comparative review on the subject^27^. Each of these force fields have their strengths and limitations. As elastic properties were included when parametrizing CSH-FF, it seems to be more suitable where mechanical properties are the focus of the study and hence it is the force field chosen for the current study. Availability of structures and force fields has spurred research in the cement and concrete community in areas such as free-energy calculations^28^, heat and mass transport properties^29–31^, polymer/C-S-H composites^32^, to just name a few.

Despite significant advances, with currently available technology, MD simulations remain constrained to small time scales on the order of microseconds^33^. As time-dependent phenomena such as creep occur on a time scale of hours, days and even years, a conventional MD simulation proves insufficient. From a probabilistic perspective, a phenomenon such as creep that occurs at longtime scales, can be viewed as a rare event process, the procession of a series of rare events^34^. In a non-equilibrium dynamic system such as a viscoelastic material under shear, the majority of the simulation time will be spent in a metastable basin. A jump to a more favorable basin by thermal activation would mean crossing an energy barrier, which requires a fluctuation with a low probability. This turns out to be the underlying cause of the disconnect between the macroscopic and nanoscopic time scales^35^. Biased molecular simulations, e.g., hyperdynamics^36^, metadynamics^37^, and Autonomous Basin Climbing^35^, are among the most noticeable time acceleration techniques. These approaches are limited to problems with small numbers of collective variables and fail for systems with soft modes. This makes their application to C-S-H rather irrelevant owing to the presence of many soft degrees of freedom in nanoconfined water molecules.

Inspired by experimental procedures in characterizing fatigue^38^ and the compaction observed in granular media due to mechanical disturbance^39^, to explore time-dependent behavior of C-S-H, we propose a simulation scheme based on the early work of Lacks and Osborne^40^. They applied incrementally increasing shear strain on a binary Lennard–Jones glass and subsequently unloaded the system after reaching a certain strain value. They observed that if the strain limit is small, the system undergoes aging and the internal energy decreases. A single cycle takes the system to a more favorable local minimum in the energy landscape. This approach artificially accelerates thermal fluctuation induced aging phenomenon. If several of these cycles are successively exerted on the sample, the system progressively visits lower minima same as those visited during a phenomenon such as creep.

In this paper, an incremental stress-marching technique is utilized to study time-dependent behavior of C-S-H. First, the C-S-H sample is loaded by cyclic perturbations where a relaxation behavior is observed. Subsequently, unloading and reloading of the sample takes place were a viscoelastic behavior is observed. The simulations show that this simple approach is able to explore different time-dependent regimes of deformation in C-S-H, ranging from creep relaxation to viscoelastic deformation. We finally investigate the impact of water content on the time-dependent behavior of C-S-H and establish its relation to the experimentally observed logarithmic creep.

## Results

### Exploring shear stress–strain relationship in C-S-H

To study time-dependent phenomenon, we create a representative structure of C-S-H. The details on constructing a realistic C-S-H model can be found in the Methods section, Supplementary Note 1 and Supplementary Figs. 1–3. The stress–strain behavior of our C-S-H models in shear return a value of 21.1 ± 2.1 GPa for the shear modulus, which compares well with a value of 21 GPa reported elsewhere^41^. Particular features are noticeable in C-S-H’s stress–strain behavior (Supplementary Figs. 4–5). First, at strains smaller than 6.5%, the shear stress–strain curve is jagged, while exhibiting a linear behavior on average. This jagged response is indicative of a rippled energy landscape with numerous local minima^42^. Upon unloading from these local minima, a small yet non-negligible residual strain appears that is in the order of 0.1%. This is indeed similar to Lacks and Osborn’s observations in binary Lennard–Jones systems^40^. Second, C-S-H exhibits a sudden increase in the residual strain in the order of 2%, if loaded beyond 1.5 GPa, which can be viewed as the yield limit. To guarantee that incremental stress marching technique (ISM) explores time-dependent phenomena rather than the failure process, we ensure that the shear stress level at all times remains below the yield limit, *τ* + *Δτ* < *τ*_*f*_ (see Supplementary Fig. 6 for a sensitivity analysis).

### Cyclic loading and unloading of the models in stages I and II

**Fig. 1:**
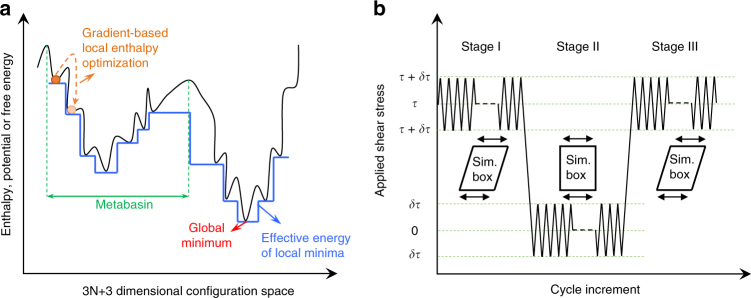
Exploring the energy landscape via the incremental stress marching approach. **a** A schematic representation of the energy landscape for a complex system. The orange ball represents the initial state of the system. Applying an external stress and local enthalpy minimization can help overcome the energy barrier, which ultimately takes the system to the minimum of the metabasin. We note that this method would not take the system to an adjacent metabasin that contains the global minimum. **b** Schematic representation of incremental cyclic scheme proposed in this study. The approach contains loading, unloading and reloading sequences, which are demonstrated from left to right. The stress is incrementally perturbed around a mean shear stress level, τ

**Fig. 2:**
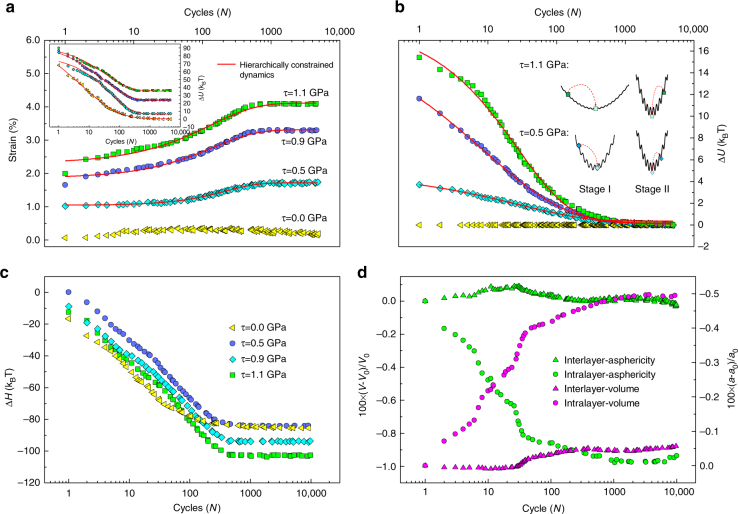
Evolution of the system under constant mean shear stress as a function of cyclic perturbations. **a** Strain evolution as cycling progresses for different stress levels. The inset shows the drop in potential energy level during the process. For the case where stress is zero, the strain remains constant, while the energy drops regardless. Such behavior is akin to relaxation phenomena in glasses and can be explained via hierarchically constrained dynamics with stretched exponential behavior. **b** The drop in potential energy during the unloading phase. We observe an exponential relaxation reminiscent of viscoelastic deformation in solids. **c** The decrease in the enthalpy as a function of cycles during the relaxation stage. This indicates that the enthalpy as well as internal energy is minimized in these relaxation processes. **d** The change in average Voronoi volume and asphericity of the atoms in the interlayer and intralayer of the specimens. We observe a significant decrease in characteristics of the Voronoi cell for atoms in the interlayer, while the intralayer attributes remain approximately constant. This highlights the reconfiguration of interlayer constituents as the primary contributor to the relaxation phenomenon at the nanoscale

A complex system such as C-S-H has a rugged energy landscape^40,43^ and the core objective of the incremental stress marching technique (see Methods section for details) is to facilitate the evolution of the system to the low-lying minima from its current state using gradient-based energy optimization as shown in Fig. 1a. Supplementary Note 2 and Supplementary Figs. 7–10 should be consulted for a simplified demonstration that the stress marching technique is equivalent to an inaccessibly long MD simulation. We subject the molecular structure of C-S-H, prepared as described in the methods section, to cyclic loading with different mean stress levels (stage I in Fig. 1b, also see Supplementary Fig. 11). As shown in Fig. 2a, this causes the structure to experience an incremental increase in shear strain, whereas the energy of the system drops to an asymptotic value in a matter of roughly 1000 stress cycles. We observe that the decline in internal energy occurs even when the mean stress level is zero. Similar behavior is observed for axial stress perturbations (Supplementary Fig. 12). To understand this behavior, we note that metastability is a characteristic of non-equilibrium systems such as C-S-H at the nanoscale. Using energy optimization, we merely obtain a structure whose energy is at a “local” minimum. However, owing to the metastable nature of C-S-H, the system is still far from the metabasin’s minimum in the energy landscape. Reaching this low-lying minimum requires overcoming a series of large energy barriers (hence, a very long simulation time). The main objective of ISM is providing a way to reach such favorable minima by a technique other than MD. Hence, each cycle of ISM, pushes the system over an energy barrier and into a new local minimum, which is more favorable than the previous one and hence lower in energy. This clearly indicates that we can view stage I as creep relaxation in shear. We note that the strain values observed here are larger than what is measured in macroscopic experiments^44^. This is due to the fact that the shear is applied parallel to the interlayer spacing and the layers can slide over each other with reduced restriction. However, in mesoscopic length scales such free movements are limited by an interconnected network of C-S-H layers. A realistic mesoscopic model would be needed to investigate such effects.

The Kohlrausch–Williams–Watts relaxation function has been extensively used as a phenomenological model to predict relaxation behavior in glass systems^45,46^. This function can be derived by several models such as hierarchically constrained dynamics^47^ or diffusion-trap^45^ and exhibits a stretched exponential shear strain as a function of cycles:

1 $$\gamma = \exp ( - (\frac{N}{{N_0}})^\beta )$$

where *N* is the number of cycles overtook, *N*_0_ can be thought of as a characteristic relaxation number, *β* is the stretching exponent and *γ* is the engineering shear strain. We find that this function is capable of accurately fitting our data. We also note that the Normalized Root Mean Squared Error (NRMSE) values lie in the interval (0.02, 0.16) for all the fitted curves in this paper. This stretched exponential relation was also proposed when modeling glass relaxation by infliction of axial tapping cyclic stress perturbations^48^.

After reaching an asymptotic value of strain and energy in the first stage, we begin stage II by unloading the system and continuing the sequence of stress perturbations. We observe that in the course of the second stage, regardless of the stress history (the mean stress applied during the first stage) all systems reach a common value of energy minimum after 5000 cycles (Fig. 2b). As we are continuously relaxing box dimensions in our structural optimizations, it is in fact the enthalpy of the system that is minimized. The decreasing trend observed for all cases in Fig. 2c corroborates this assertion. The distinction between enthalpy and potential energy evolution proves more interesting for the third stage as described in the next section.

The results in Fig. 2a–b, can be understood in an enthalpy landscape framework, similar to the approach adopted to explain protein folding in biological systems^49^. A rugged enthalpy landscape can be envisioned for the system as seen by the schematic inset in Fig. 2b. Initially, the system resides in a metastable state, which is separated from the minimum of the basin by numerous small barriers. Application of external stress distorts the enthalpy landscape of the system and lowers the secondary barriers in the basin that would ultimately translate to higher transition rates^50^. Malandro and Lacks also showed that application of external shear strain causes the local minima of the potential energy surface to disappear, leading to mechanical instabilities that force the system into unexplored regions of the energy landscape. As seen from the inset of Fig. 2b, the landscape is transformed again upon unloading and the system is no longer in the minimum of the metabasin. Further cycling will again result in a drop in the internal energy to the lowest lying point in the metabasin, as observed in the inset.

To further understand the kinematic sources of relaxation in the C-S-H sample, Voronoi volume and the asphericity of the Voronoi cells were investigated for each atom. Asphericity is defined as the radius of the sphere with the same volume as the atom’s Voronoi cell^51^:

2 $$\eta = \frac{{A^3}}{{36\pi V^2}}$$

where *A* is the total area of the convex hull of the Voronoi cell and *V* is the Voronoi volume of the cell. At this stage, we distinguish between atoms in the defective intralayer calcium–silicate sheets and those forming the interlayer spacing. We observe a significant decrease in both the average asphericity and Voronoi volume of the interlayer constituents, interlayer calcium, hydroxide, and water molecules, whereas the values for the intralayer atoms remain constant (Fig. 2d). This can be regarded as evidence that the interlayer species are the main contributors to the time-dependent relaxation of C-S-H globules under constant stress.

### Cyclic reloading of the models in stage III

**Fig. 3:**
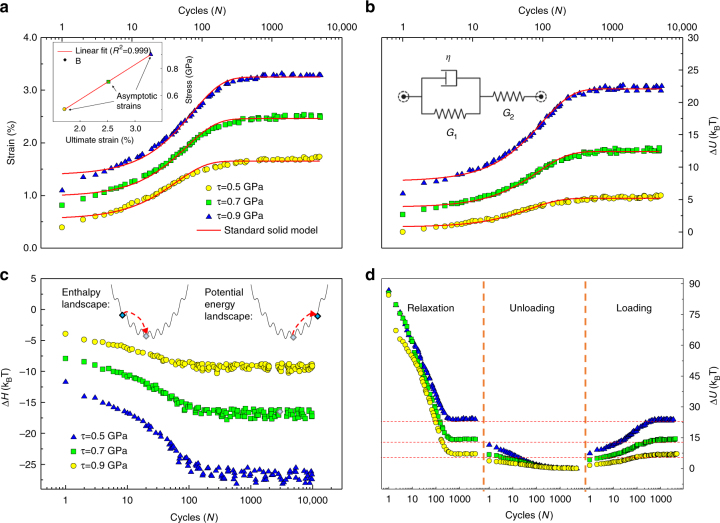
Time-dependent characteristics of the reloading stage. **a** Strain as a function of the number of cycles for three different stress levels. The inset shows that the asymptotic value of strain reached at each stress level scales linearly with the applied shear stress. **b** Increase in the internal energy of the system as a function of the number of cycles. The inset shows the standard solid model used to fit the atomistic simulation data. **c** The decrease in enthalpy as a function of the number of cycles, regardless of the applied stress level. The inset schematically represents the evolution of the system in both the enthalpy and potential energy landscapes. While the potential energy of the system increases by climbing the energy landscape, the enthalpy decreases gradually to achieve thermodynamic equilibrium state. **d** A holistic view of the loading, unloading and reloading sequences that signifies the irreversibility of the relaxation process and reversibility of the viscoelastic behavior

If external stress is applied to the system at the end of the unloading stage, an instant increase in energy and shear strain is observed. Moreover, continued cyclic perturbations will cause the energy and strain of the system to further increase as shown in Fig. 3a–b. This form of cycle-dependent behavior is akin to that of viscoelastic behavior of solid materials. We verify that the final asymptotic strain scales linearly with the applied stress, see inset of Fig. 3a. Consequently, we use the phenomenological standard solid model to fit our data. This model, as shown in the inset of Fig. 3, is composed of two spring elements and a damper. Solving the differential equation governing this model under constant stress, *τ*_0_, leads to:

3 $$\gamma = \frac{{\tau _0}}{{G_2}}\left\{ {\frac{{G_1 + G_2}}{{G_1}} - \mathrm{e}^{ - \lambda N}} \right\}$$

where *G*_2_ and *G*_1_ are the instantaneous stiffness and damping stiffness of the elastic components, *λ* = *G*_2_*/μ*_0_, and *μ*_0_ is proportional to the viscosity of the material. We obtain a value of *G* = 28.8 ± 1.3 GPa for the average of the resultant shear modulus. Although this has the same order of magnitude as those calculated in direct shear (21.1 ± 2.1 GPa), the discrepancy is non-negligible. This can in part be attributed to the approximate linear relationship that is assumed between time and number of cycles.

To further understand the observed viscoelastic phenomenon, rugged enthalpy and energy landscapes can be imagined for this complex system (inset of Fig. 3c). As the specimen is loaded, the system is seen climbing from the minimum of the basin to higher levels of potential energy. If we load a standard solid, we will observe the same behavior. This might seem counter-intuitive; however, a glance at the evolution of the system enthalpy (Fig. 3c) shows a decrease as stress perturbations continue. This means that with each cycle the system is moving to a more favorable minimum in the enthalpy landscape. Viscoelasticity also asserts that the system should recover the initial configuration and energy without any residual deformation. This is proven to be the case for C-S-H, as we note that in Fig. 3d the final energy state in stage I coincides with the final energy state in stage III.

### Intraglobular to interglobar transition

**Fig. 4:**
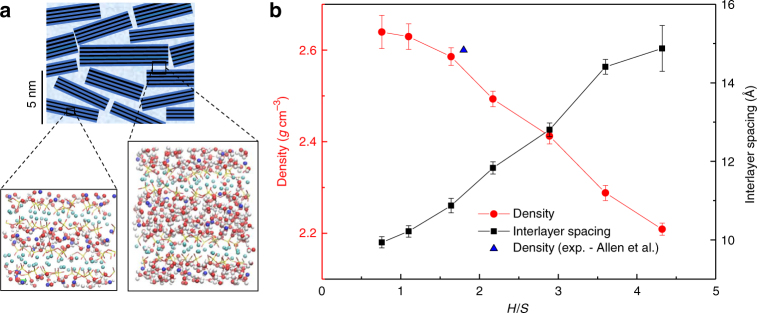
The impact of the nanoconfined water content on the morphology, structure and density of C-S-H. **a** Schematics of C-S-H globules at the mesoscale shows how atomistic models with varying interlayer water content can potentially correspond to the inter and intraglobular behavior of C-S-H. The black strips represent silicate layers, dark blue is the intralayer or adsorbed water and light blue is the water between nanoglobules of the C-S-H. In the atomistic models, red represents oxygen, blue is interlayer calcium, cyan shows intralayer calcium and white and yellow represent respectively hydrogen and silicon atoms. **b **Variations of density and interlayer spacing as a function of *H*/*S* and comparison with Small Angle Neutron Scattering density measurements

The exponential viscoelastic behavior observed so far differs from the logarithmic creep observed in nanoindentation experiments carried out by Vandamme and Ulm^52^, where nanogranular behavior of C-S-H is cited as the origin of creep. According to nanogranular theory, C-S-H mesotxture can be viewed as the assembly of colloidal C-S-H nanoparticles of imperfect tobermorite-like minerals^11^. This theory was hypothesized on the basis of observations obtained from small angle X-ray and Neutron scattering as well as statistical nanoindentation experiments^53–55^. A simultaneous look at the experimental results and those obtained by atomistic simulations for models with different water content, raises the question: is creep caused by sliding of calcium–silicate layers over each other as envisioned by the Feldman–Sereda model^10^ or by the nanoglobular mesoscopic behavior. To address this question, we try to reproduce the sliding of C-S-H layers by investigating samples with different water content in the interlayer. In fact, inter and intraglobular behavior of C-S-H can be potentially explored by merely adjusting the number of interlayer water molecules as shown by the atomistic simulation boxes in Fig. 4a. Note that in the current study, intraglobular water and interlayer water denote the same constituents and can be used interchangeably. Here, we choose seven samples with varying water-to-silicon molar ratio (*H/S*), Fig. 4b. These samples are prepared simply by introducing more water molecules in the interlayers of the C-S-H model used for previous sections. We find that although the interlayer spacing increased linearly with water content, the density decreases linearly with it. We also investigate the behavior of amorphous ice, the structure of which can be obtained by quenching a thermal simulation box of water using structural optimization (infinitely fast). The amorphous ice sample can be regarded as the limiting case for the C-S-H water content (*H*/*S* → ∞).

**Fig. 5:**
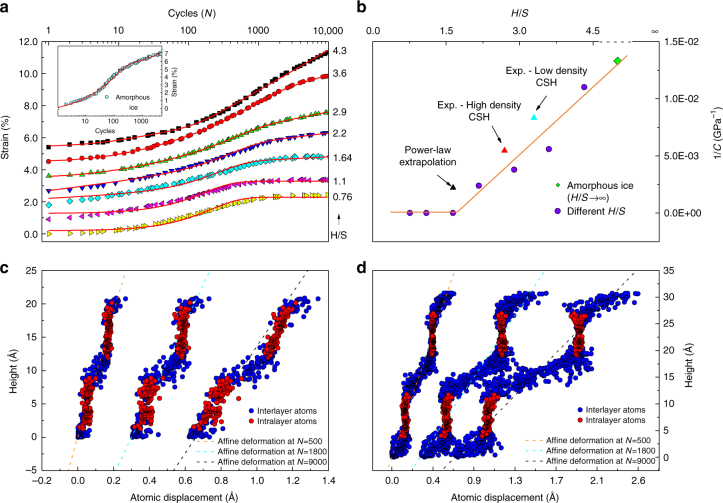
The impact of nanoconfined water content on the time-dependent properties of C-S-H during reloading stage. **a** A logarithmic component gradually appears as the water content increases. The inset shows the same experiment carried out on a box of amorphous ice. The graphs have been shifted for clarity.** b** Variation of creep compliance with the nanoconfined water content and their direct comparison with experimental values^52^ for the high and low density C-S-H using nanoindentation technique. A transition is observed in the region, where intraglobular water appears. We can also see how the values converge to the case of a box of amorphous ice in the limit. **c**, **d** Horizontal deformation of the sample with height for a sample with low water content in (**c**) and high water content in (**d**). While at high *H*/*S* ratio the layers freely slide over each other without distorting the solid constituents, the sliding forces at lower *H*/*S* content interlocks adjacent layers that promotes the shear deformation of the solid parts. This implies that the design of non-aging C-S-H materials is closely tied to molecular manipulation of interglobular interlocking structural motifs

We first fully relax these eight models by subjecting them to 10^4^ stress cycles and subsequently reload them to monitor their behavior during stage III (Fig. 5a). For samples with low-water content, a purely exponential viscoelastic behavior is observed. However, as the water content increases, a logarithmic non-asymptotic component emerges. These results show a transition from a purely exponential viscoelastic behavior to a mixed exponential-logarithmic one. To capture this behavior quantitatively, we propose the following phenomenological function for samples with higher water content:

4 $$\gamma (N) = \frac{{\tau _0}}{G}\left( {1 - \exp \left( { - \frac{N}{{N_0}}} \right)} \right) + \frac{{\tau _0}}{C}\log \left( {1 + \frac{N}{{N_0^\prime }}} \right)$$

where 1/C and *N*_0_ are, respectively, creep compliance and characteristic number of stress perturbations. The exponential part of this equation is inspired by Eq. (3) and the logarithmic part can be interpreted according to experimental observations in nanoindentation tests^52^. The direct measurement of *H/S* at the mesoscale proves difficult. Here, we estimate *H/S* for experimental data based on the reported packing fraction, *η*, as follows:

5 $$\left( {\frac{H}{S}} \right)_{{\mathrm{total}}} = \left( {\frac{H}{S}} \right)_{{\mathrm{CSH}}} + \frac{{(1 - \eta )\rho _{\mathrm{w}}m_{{\mathrm{sim}}}}}{{M_{\mathrm{w}}n_{{\mathrm{Si - sim}}}\eta \rho _{{\mathrm{CSH}}}}}$$

where *ρ*_w_ and *ρ*_C-S-H_ are, respectively, the density of water and C-S-H. *m*_sim_ is the mass of the simulation box in g.mol^−1^, *n*_Si-sim_ is the number of silicon atoms in the simulation box, and *M*_*w*_ denotes the molecular weight of water in g.mol^−1^. We find that creep compliance measured for low and high density C-S-H in nanoindentation experiments is close to calculations from our atomistic simulations, Fig. 5b. The transition of behavior from purely exponential to a combined logarithmic-exponential brings us to the conclusion that each individual globule behaves viscoelastically. However, their sliding over each other, which is modeled by a larger separation of layers with interglobular water, introduces a logarithmic behavior. In other words, these results confirm the hypothesis that creep of hydrated cement originates from reconfiguration of C-S-H globules at the mesoscale rather than sliding of individual C-S-H sheets over each other.

To get a better understanding of the underlying cause of the transition of behavior from exponential to logarithmic, deformation profiles are plotted in Fig. 5c–d for specimens with different water content at several stages during the cycling process. It can be clearly observed that for the case with higher water content (*H*/*S* > 1.7), the layers can easily slide over each other and there is a negligible shear deformation in the solid part of the C-S-H layer and the deformation only arises from the interlayer constituents. For the case with lower water content (*H*/*S* < 1.7), a frictional interlocking exists between the motifs of the layers and larger energy barriers should be overcome for sliding to happen. This observation is in agreement with a decreasing trend observed previously for elastic constants as a function of *H*/*S*^56^. The energy barriers and their dependence on the interlayer spacing have been recently studied by Masoumi et al.^57^. Coordinate files for the initial structure before loading and after loading can be viewed as Supplementary Data 1 and 2, respectively. These data are for the case *H*/*S* ≈ 1.7.

## Discussion

In this paper, stress-induced time-dependent behavior of C-S-H was investigated by application of a chain of aging shear cycles at the nanoscale. We showed that if a C-S-H atomistic model is subjected to repeated aging/rejuvenation cycles under a constant shear stress, the potential energy of the system will decrease until an asymptotic value is reached. This is akin to relaxation in glassy systems and can be explained using stretched exponential hierarchically constrained dynamics model. Voronoi and asphericity analysis of the model during relaxation show that shear relaxation of C-S-H is rooted in reconfiguration of interlayer constituents. In addition, reloading the specimen after the relaxation stage will result in an increase in the potential energy of the system. We show that in spite of this increase, enthalpy still decreases, which means the system is moving to more favorable minima in the enthalpy landscape. We also demonstrate that this stage can be well described by a three-component standard solid model, akin to an exponential viscoelastic behavior. We observe a transition window, where the behavior changes from purely exponential to combined logarithmic-exponential behavior with increasing interlayer water content. A combined logarithmic-exponential function was also proposed for a phenomenological description of the observations. An in-depth analysis of shear deformation at the atomic level demonstrated the effectiveness of the shear locking mechanism as a potential venue to restrain non-asymptotic time-dependent behavior.

Despite the abovementioned striking observations using a rather simple approach, this work can benefit in many ways from future research at the intersection of cement and concrete science, molecular simulation, and statistical physics. Considering the complexity in modeling cementitious materials, we note that using a non-reactive classical force field limits our simulations to nonaging creep. Investigation of aging creep, i.e., creep associated with chemical reactions^58^ (breakage and formation of covalent bonds and deformation induced dissolution–precipitation mechanisms), can be conducted using a reactive potential in conjunction with the ISM. This could be computationally prohibitive, owing to the large number of enthalpy optimizations and the associated cost of employing reactive potentials. This motivates further research toward devising algorithms to reduce the cost of reaching a low-lying minimum. Furthermore, to truly test the observed intraglobular to interglobular transition behavior, prohibitively large atomistic models would be needed. This calls for realistic upscaling of C-S-H atomistic models through proper coarse-graining techniques and applying ISM to the model at the larger length scale. Regarding composition of C-S-H, one expects fewer bridging silicate tetrahedra to be present at higher calcium to silicon ratios (C/S). This means that the barriers owing to steric repulsion will be altered significantly and time-dependent behavior could possibly be affected. Moreover, previous research has shown that the number of hydrogen bonds increases with C/S, which alters the interlayer hydrogen bond network^31^. A separate study should be carried out to unravel the role played by composition.

From statistical physics standpoint, presence of small energy barriers^59^ in a complex system such as C-S-H limits the applicability of existing accelerated dynamics techniques. A future progress in this field can potentially unlock the true dynamic trajectory of long-term time-dependent response of structural materials. Assuming that the transition state theory is applicable for studying C-S-H time-dependent behavior^60^, the height of barriers separating two inherent structures, i.e., local minima, governs transition rates. Measuring the energy barriers for C-S-H, using methods such as Nudged Elastic Band^61^, proves futile due to the presence of many soft modes in nanoconfined water. This means that establishing a link between time and number of cycles in ISM would only be qualitative at best. Supplementary Note 2 and Supplementary Figs. 7–10 discuss a derivation using a simplified 1D synthetic energy landscape. However, similar results can be obtained more rigorously using higher dimensional SELs such as NK or transition matrix models^62^.

Altogether, the results of this work shed more light on various aspects of fundamental mechanisms that cause time-dependent behavior in C-S-H. Furthermore, acceleration techniques such as the proposed ISM would bridge the existing gap between time scales in atomistic simulations and nanomechanical testing of condensed phases, which would ultimately pave the way for rational design of construction materials, and other glassy systems such as oxide and metallic glasses with reduced aging characteristics.

## Methods

### Molecular structures

C-S-H, the hydration product that is responsible for cement’s strength and durability, is a quasi-glassy material with an average C/S around 1.7^8,63^. In this paper, we use a slightly modified version of the model proposed by Abdolhosseini Qomi et al.^64^, as it closely captures several key experimental observations such as density, basal spacing and XRD patterns (Supplementary Fig. 2)^54,64,65^. Similar atomic models of C-S-H can be constructed by following different procedures as carried out, for instance by Kumar et al.^66^ or Kovačević et al.^67^. For more simulation details, Supplementary Note 1 and Supplementary Figs. 1–3 should be consulted. Herein, the atomic structure of C-S-H is rationalized through structural modifications of Hamid’s 11 Å tobermorite^68^, a crystalline mineral with a layered structure composed of infinite dreierketten silicate chains with C/S = 1. To construct realistic molecular models of C-S-H akin to those observed in modern hydrated Portland cement, we randomly remove bridging and (occasionally) pairing sites from tobermorite’s infinite silicate chains. This method of constructing the molecular structure of C-S-H is shown to provide models that are in close agreements with ^29^Si NMR^69^, Inelastic Neutron Scattering^54,70^, normal and high-pressure X-Ray diffraction measurements^55^. The final composition reached is Ca_1.7_Si_1.0_O_2.5_(OH)_1.7_(H_2_O)_0.9_. Note that to study intraglobular to interglobular behavior, the amount of water in this formula is varied to observe different regimes of time-dependent behavior. The literature can be consulted for further detail on model construction^9,64,65^.

### Force field and simulation details

Here, we use transferable CSH-FF potential^24^ designed particularly for cement hydrates. This ClayFF-based force field^23^ is shown to reproduce characteristics of C-S-H ranging from mechanical to thermal properties^30,31^. ReaxFF (a bond-order based potential) is an alternative force field for cementitious materials that possesses the ability to capture bond breakage/froming during deformation^41^. However, its high computational cost makes it impractical for the current study. We use LAMMPS simulation package for all computations^71^. All models in our numerical experiments are initially relaxed in the NPT ensemble using Nose-Hoover thermostat^72^ at 300 K and Parinello-Rahman barostat^73^ at 1 atm for 2 ns and time steps of 1 fs. After proper volumetric relaxation by inflicting 1000 cycles of volumetric stress perturbation of 0.1 GPa, we confirm a density of 2.59 g/cm^3^ in close agreements with SANS measurements^54^. Also we repeated the simulations for a system twice as large to observe the impact of size effects (Supplementary Fig. 3).

### Incremental stress marching technique

Here, we envision a three-stage cyclic scheme that applies successive stress perturbations with varying average stress levels, which are kept within the elastic limits of the material. A complex system such as C-S-H has a rugged energy landscape^40,43^ as discussed in the results section and the core objective of this technique is to facilitate the evolution of the system to the low-lying minima from its current state using gradient-based energy optimization as demonstrated in Fig. 1a. Supplementary Note 2 and Supplementary Figs. 7–10 should be consulted for a simplified demonstration that the stress marching technique is equivalent to an inaccessibly long MD simulation.

Our approach continuously monitors the internal energy, enthalpy, stress, strain, and atomic trajectory in molecular models during loading (stage I), unloading (stage II), and reloading (stage III) phases, as demonstrated schematically in Fig. 1b. Strain is calculated as explained in Supplementary Note 3. In each individual stress cycle, the stress is perturbed about the mean value with increments of 0.01 GPa and an enthalpy minimization is carried out at the end of each increment. This is justified owing to the negligible effect of the entropic term in Gibbs free energy for a solid system such as C-S-H (*G* = *H* − *TS* where *G*, *H* and *S* are the Gibbs free energy, enthalpy and entropy of the system, also see Supplementary Note 4).

A threshold of ± 0.1 GPa is defined around the mean value and the loading direction is reversed when the limits of the envelope are reached. We use a total of 3 × 10^4^ stress cycles in our ISM framework, which would ultimately lead to about 2 × 10^6^ minimization steps. This staggering number of enthalpy minimizations signifies the infeasibility of eigenvalue-based minimizers that ensure convergence to a local minimum. However, unlike stress tapping approach^74^, our incremental updated Lagrangian method resolves an artifact of gradient-based minimizers, i.e., the possibility of converging to a saddle point. We note that the ISM technique is computationally expensive due to millions of enthalpy minimizations required in the process.

### Model dependency

First, we note that the results presented in this paper, have been obtained based on a model prepared via a similar procedure as explained in Abdolhosseini Qomi et al.^9^. However, there are other more ordered models available in the literature such as those proposed by Kumar et al.^66^ and Kovačević et al.^75^. We have applied ISM to these models and also a completely crystalline sample of tombermorite and a similar behavior as the model used herein was observed (Supplementary Figs. 13–17 and Supplementary Note 5). This points to the fact that the layered structure and interlayer spacing can be taken as the main contributors to the time-dependent behavior of C-S-H and other minor structural modifications do not change the overall behavior.

### Data availability

The authors declare that the main data supporting the findings of this study are available within the article and its Supplementary Information files. Extra data are available from the corresponding author upon request.

## Electronic supplementary material

Supplementary Information

Description of Additional Supplementary Files

Supplementary Data 1

Supplementary Data 2
