# Supplementary material for: Nanoscale origins of creep in calcium silicate hydrates
Source: Nat Commun. 2018 May 3;9:1785. doi: 10.1038/s41467-018-04174-z (PMC5934396; doi:10.1038/s41467-018-04174-z)
Supplement: Supplementary file 1 — Supplementary Information [file 41467_2018_4174_MOESM1_ESM.pdf]

Supplementary Information

Nanoscale Origins of Creep in Calcium Silicate Hydrates

By: Morshedifard *et al.*

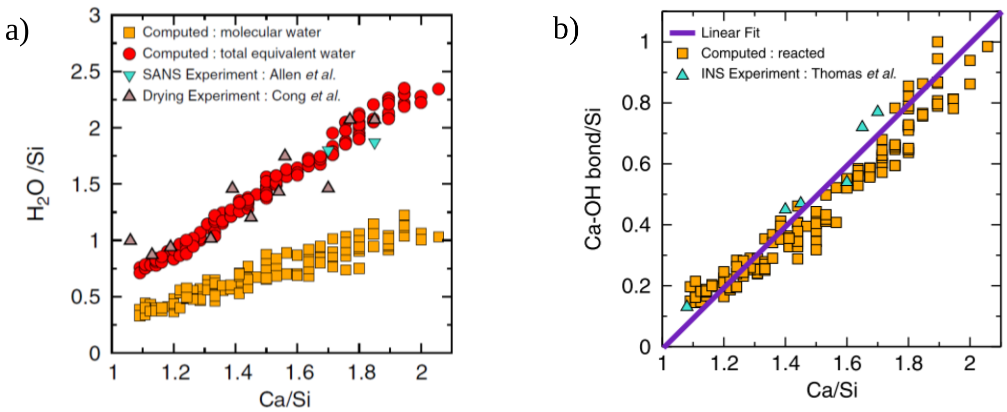

Supplementary Fig. 1. Different compositions of C-S-H a) Variation of molecular and equivalent water content in C-S-H computed from reactive simulations b) Variation of Ca-OH bonds with Ca/Si ratio. (adapted from Abdolhosseini *et al*[1])

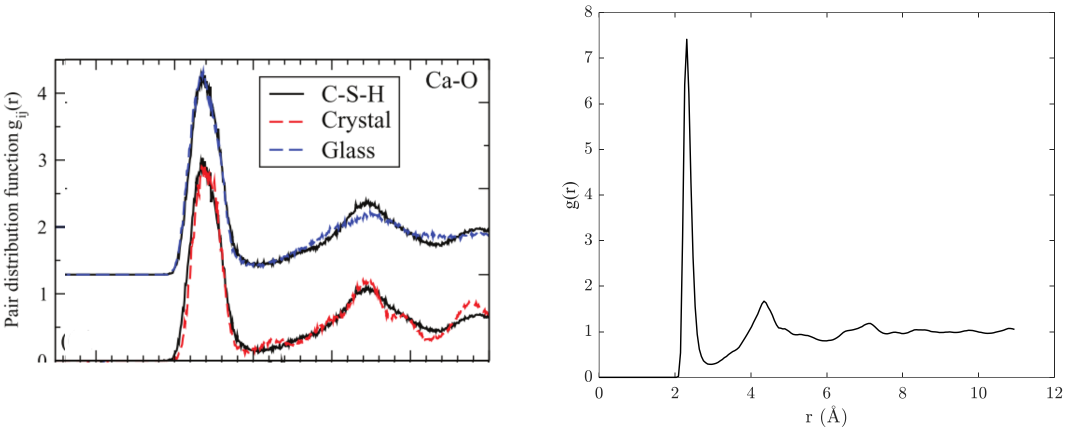

Supplementary Fig. 2: partial pair distribution functions for Ca-O pairs. a) the model used in Abdolhosseini *et al.*[1]. b) the model used in the current study.

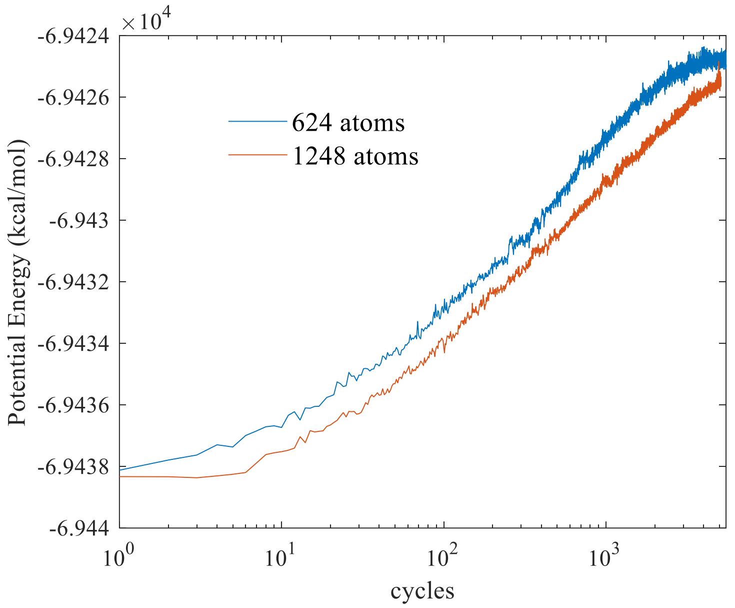

Supplementary Fig. 3: Comparison between the energy curves of the third stage for systems with 624 and 1248 atoms.

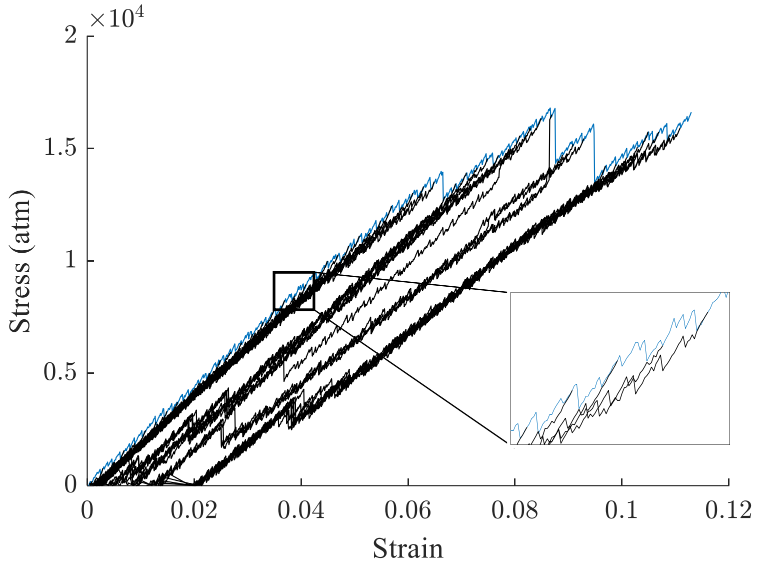

Supplementary Fig. 4: Shear loading of the specimen and unloading at different strain values. An elastic behavior is observed up to a stress of about 15000 atm. We have unloaded the system at different strain values. It is clearly observed that up to a stress level of about 15000 atm, the system shows negligible residual strain upon unloading. Perturbation values should be chosen in a way that the system remains below the yield stress.

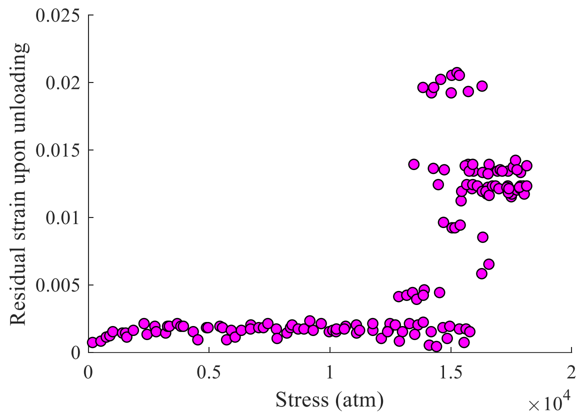

Supplementary Fig. 5: Residual strain as a function of the stress value where unloading was initiated. The onset of inelastic behavior is observed to be around 15000 atm.

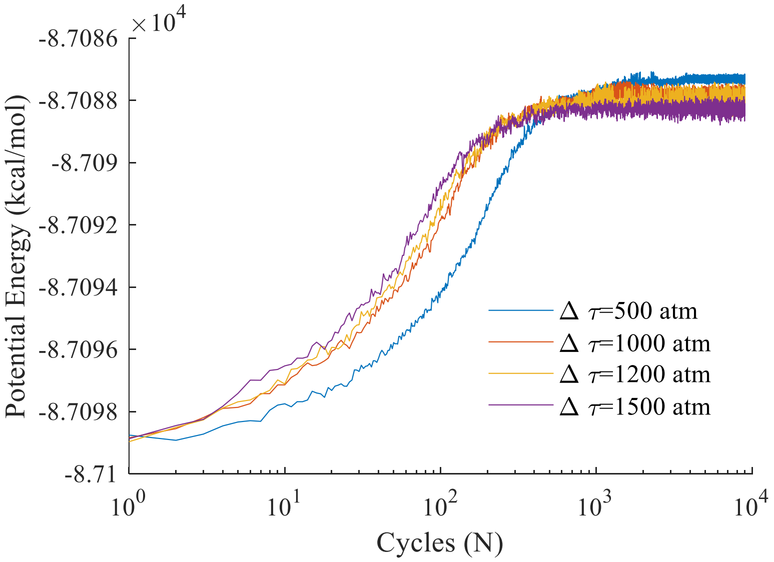

Supplementary Fig. 6: Loading of the specimen with different $\Delta\tau$ values. A similar asymptotic behavior is observed similar to those demonstrated in the manuscript. To demonstrate that the viscoelastic behavior observed in the text is not dependent on the choice of $\Delta\tau$, we have run the simulations for four different. We can see that the general behavior of the material is not altered as $\Delta\tau$ changes.

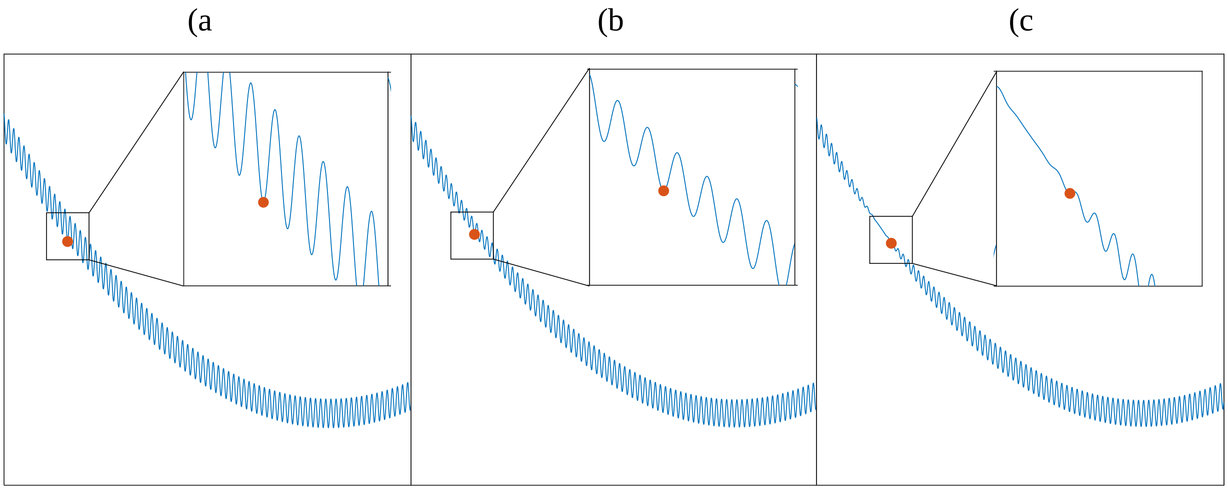

Supplementary Fig. 7: From left to right: The gradual distortion of the enthalpy landscape due to gradual increase in F in the schematic 1D model. a) initial state b) distorted state c)a transition to the next state takes place.

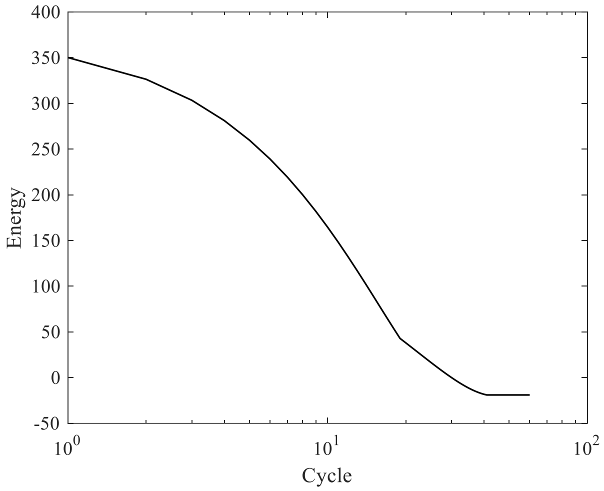

Supplementary Fig. 8: The decrease in energy as oscillations are applied to the 1D SEL.

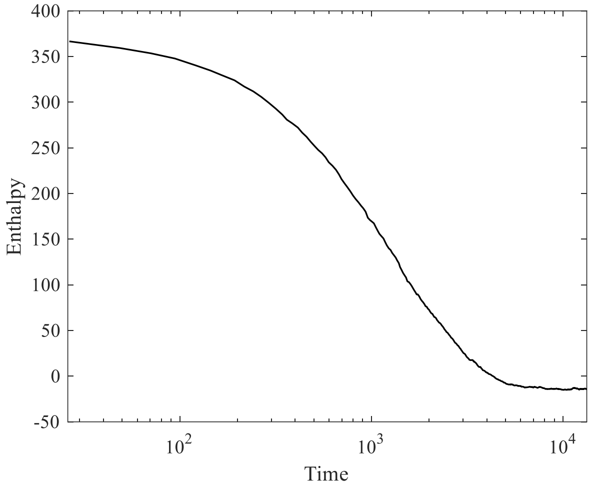

Supplementary Fig. 9: Energy drop as a function of time from kinetic Monte Carlo simulations on the 1D SEL model.

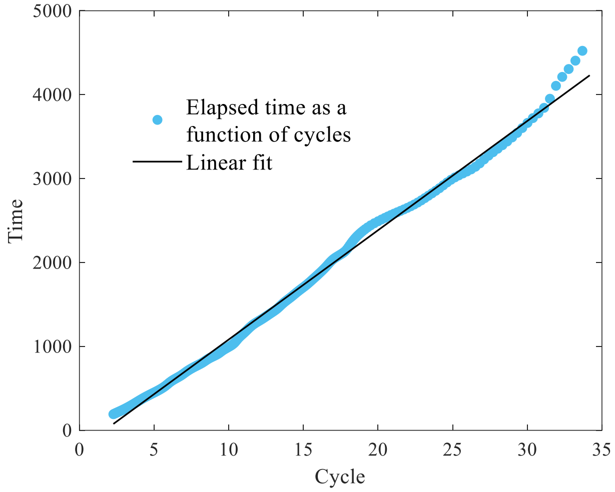

Supplementary Fig. 10: The time-cycle relationship after combining the results of Monte Carlo and ISM simulations for the 1D SEL model considered herein. A linear relationship is observed.

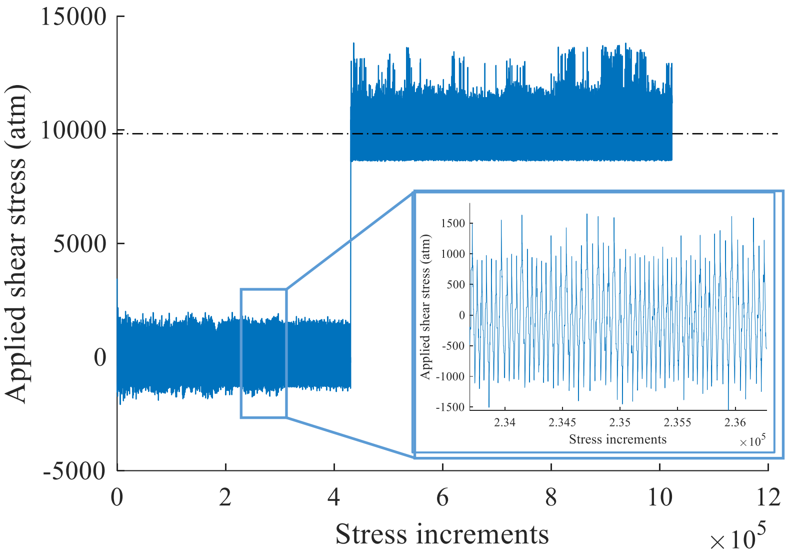

Supplementary Fig. 1: Gradual stress perturbations. A seamless transition to the next cycling stage (mean shear stress of 10000 atm) can be observed. As explained in the text, the Incremental Stress Marching technique, consists of load cycles on the specimen with a predetermined average stress. This sample is initially loaded at 0 stress and stress perturbations of $\Delta\tau = 1000$ atm are applied. The periodic nature of these stress cycles are demonstrated more clearly by maginification of a portion of the graph.

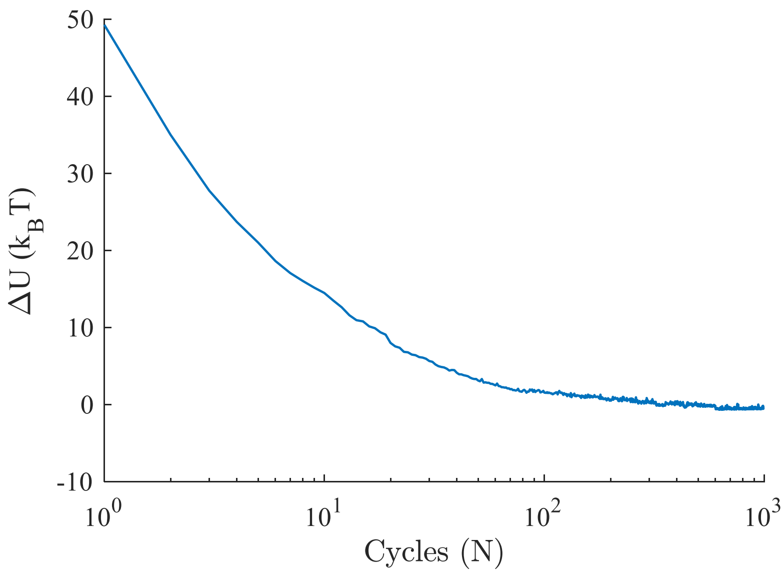

Supplementary Fig. 2: Decrease in potential energy as a result of cyclic perturbations on the system in an axial direction. Inflicting cycling stress perturbations to the system in the normal direction, causes a similar decrease in energy as Fig. 2 in the main text. This similarity is expected since cycling at zero stress in any direction signifies relaxation.

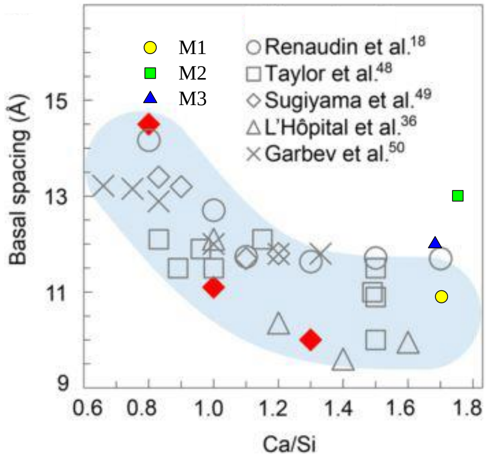

Supplementary Fig. 13: A comparison of basal spacing values for the three models considered and those of experiments.

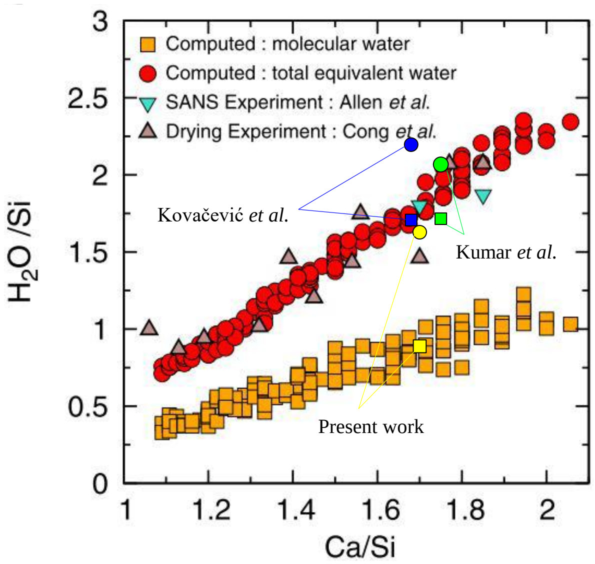

Supplementary Fig. 14: Variations of total equivalent and molecular water with C/S. Points corresponding to different models can be observed

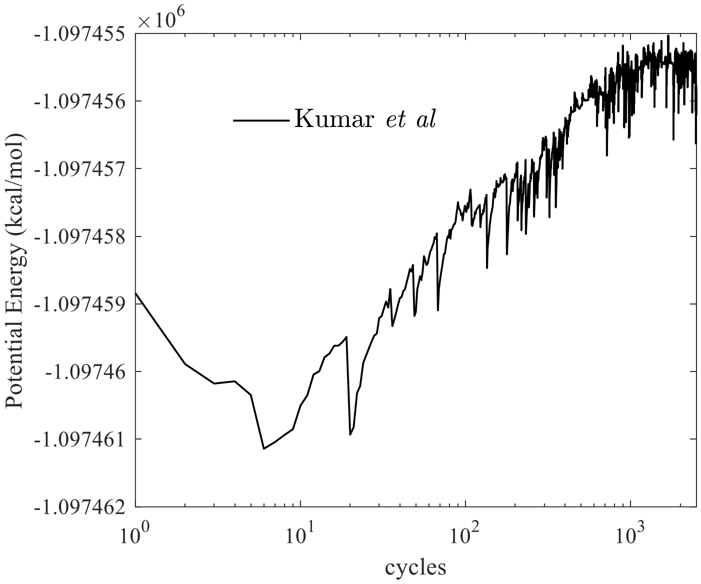

Supplementary Fig. 15: Observed behavior due to application of ISM/A in the loading stage for the model proposed by Kumar *et al*[2].

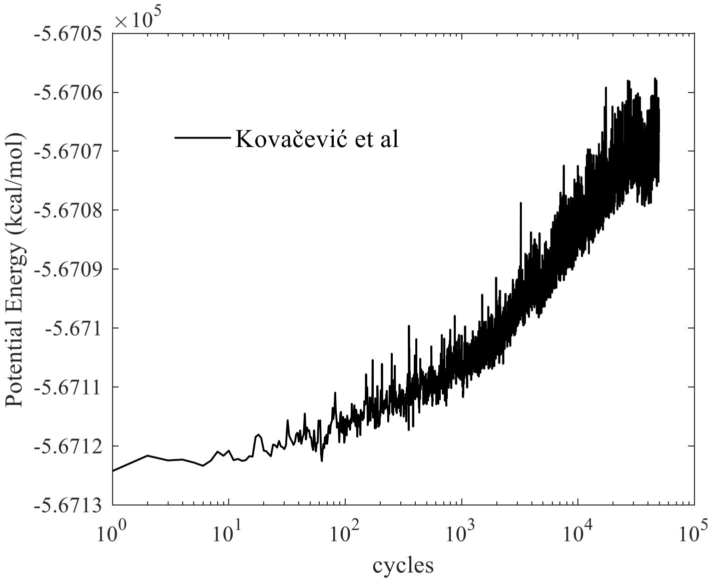

Supplementary Fig. 16: Observed behavior due to application of ISM/A in the loading stage for the model proposed by Kovačević *et al*[3].

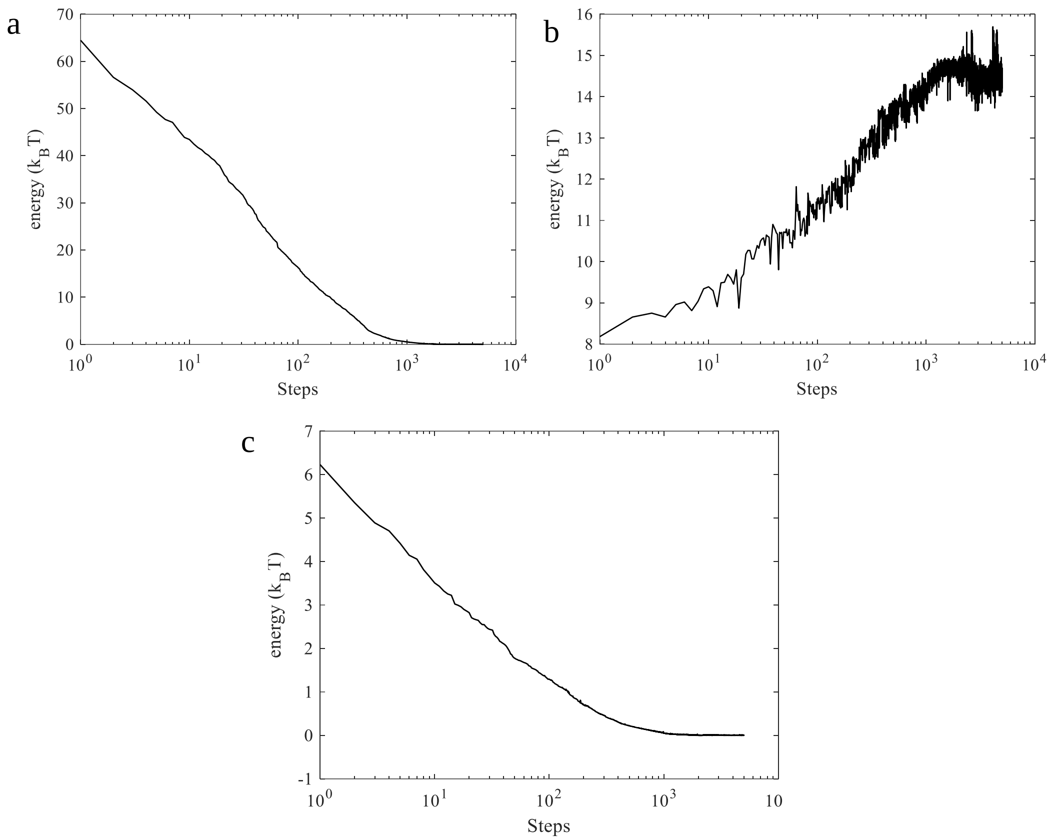

Supplementary Fig. 17: ISM applied to 11Å Hamid's tobermorite (a) The relaxation stage under $\tau = 0$ GPa (b) loading to $\tau = 0.75$ GPa and (c) unloading the specimen following the loading stage.

Supplementary Note 1

To make a structure that captures the experimental aspects of the CSH that exist in ordinary Portland cement, we start from the structure of its natural analogue, 11 Å Tobermorite[8]. To avoid finite size effects a $4\times4\times1$ supercell was constructed ($a=13.272$ Å, $b=24.436$ Å, $c=24.435$ Å, $\alpha=89.684^\circ$, $\beta=90.125^\circ$ and $\gamma=123.208^\circ$). Next, calcium to silicon ratio is increased by removing the bridging sites (charge neutral SiO_2) from the infinite silicate chains of tobermorite. Note that based on NMR experiments, such removal should produce only dimers, pentamers, etc in compliance with the dreierketten rule[9]. We note that after full relaxation of the prepared structure using CSH-FF forcefield[10], we have detected a trimer in the final structure. This can be attributed to an artifact of the potential set used and although comparison with other forcefields show that such artifact does not impact the conclusions of this paper, care should be taken when using this structure in more demanding simulations.

When all the bridging sites are removed, C/S stands at 1.5. Further increase in C/S is achieved by removing pairing sites from the structure until a C/S=1.7 is reached which is the average value observed in experiments[11]. Next, to charge balance ending oxygen atoms with hydrogen atoms, we use the results of reactive simulations carried out by Abdolhosseini *et al* as shown in Supplementary Fig. . The model so prepared has 48 Ca-OH bonds, 56 SiOH bonds and 50 water molecules. The final structure contains 634 atoms and the empirical formula is: $Ca_{1.7}Si_{1.0}O_{2.5}(OH)_{1.7}(H_2O)_{0.9}$. The coordinates of the relaxed structure can be obtained as additional supplementary material to this paper.

To model the intraglobular to interglobular (ITI) behavior in the simulations, we gradually increase the water content in the interlayer of the model prepared initially by the previously mentioned procedure. Naturally, the basal spacing needs to be increased to maintain the proper density for water.

We use the LAMMPS package to run the simulations, Ewald summation was used to account for long range electrostatic interactions. Also, a cutoff of 12 Å was chosen for non-bonded interactions. We used Nosé Hoover barostats and thermostats for rescaling pressure and temperature in different ensembles.

The forcefield. Clay-FF is one of the main forcefields available for modeling complex hydrated inorganic minerals such as clay and tobermorite[12]. However, it has known deficiencies in reproducing the correct elastic properties for cementitious materials. Shahsavari *et al*[10] show a clear comparison between the predictive powers of CSH-FF and ClayFF.

CSH-FF is a ClayFF-based forcefield that incorporates second order properties (i.e. elastic moduli) in the fitting process which makes it the more accurate forcefield when it comes to silicate minerals prevalent in cementitious materials. Consequently, we have utilized CSH-FF in all our simulations. In this forcefield, harmonic linear bonds are defined for the SPC water molecules and hydroxyl groups. Also, we have angular harmonic contributions to energy from water molecules. All the other interactions are captured by non-bonded Lennard Jones and electrostatic potentials.

Considering the fact that CSH-FF is also capable of accurately reproducing the cell parameters for cementitious materials, it seems to be the most suitable forcefield available for our purposes.

Calcium coordination. To compare the model used in this study and the previous models used in Abdolhosseini et al., we compare the partial pair distribution function for Ca-O pairs in Supplementary Fig. .

It is clearly observed that the peaks are narrower in the model used for the current study which indicate a higher degree of order. Moreover, we calculate the coordination of each calcium atom by integrating the PDF up to the first minimum as follows:

$$n(r) = 4\pi\rho\int_0^{r'} g(r)r^2 dr$$

where r' is the position of the first minimum. We obtain a value of 5.93 for average coordination number and the minimum coordination of Ca atoms stands at 4.8. Considering the high C/S of the sample, we believe this to be a satisfactory check for the model.

Finite size effect. To make sure that the observed behavior in this paper, is not affected by the finite size of the specimen used, we also ran the simulations for a system twice as large as the one used for calculations. A comparison of the results as shown in Supplementary Fig. demonstrates the adequacy of the system size chosen for the calculations. The difference between the energies stands at 0.001%.

Supplementary Note 2

Time-cycle relationship. A rigorous derivation for time-cycle functional form would be possible if the enthalpy landscape for C-S-H was available. Even then, finding the inherent structures (basins, minima) traversed during dynamics would be challenging due to high mobility of water molecules and presence of numerous small barriers[4]. We believe a plausible solution is to replace C-S-H with a synthetic enthalpy landscape (SEL) that possesses similar features as the actual landscape and simulating both dynamics and ISM based on the synthetic model. This approach has been applied previously to understand behavior of glassy systems under shear strain by using toy models such as the NK or transition matrix models[5].

For our purposes, the NK model seems to be the most promising. However, two main issues should be resolved to obtain the inherent structures for conventional dynamics and ISM:

1- available toy models introduce a parameter equivalent to strain in the potential energy expression for the system. However, stress is perturbed in ISM and a new strategy is needed to accommodate that. In other words, an enthalpy term should be optimized rather than potential energy.
2- An accurate dynamics should be defined for configurations in these toy models (we have a discrete configuration space for the NK model).

The current paper is mainly focused on time-dependent behavior of C-S-H and a comprehensive discussion on ISM would be out of scope of the article. However, using a simplified 1D analogue of our system's enthalpy landscape, we try to demonstrate equivalency of conventional dynamics and ISM.

Guided by the behavior observed by ISM when applied to C-S-H as discussed in the paper and the underlying ruggedness of the enthalpy landscape, we assume the following 1D model for the enthalpy of the synthetic system:

$$H = \alpha x^2 + \beta \sin(\omega x)(1 - \frac{F}{\sqrt{(1+\zeta(x-u)^2)}}) \tag{1}$$

where α, β, ω and ζ are constants. F plays the role of force and u serves two purposes: 1- it implicitly adds the effect of absent dimensions to the 1D representation 2- Keeps the **change** in barrier heights close to the current configuration symmetric and constant for ISM cycles.

We first test this model by performing gradual cyclic oscillations ($F \pm \Delta F$) while performing optimizations after each increment. Three intermediate stages are displayed in Supplementary Fig. . The enthalpy landscape is gradually distorted until a single or multiple jumps to adjacent minima occur.

The cyclic loading is repeated until the system reaches the minimum of the metabasin. As in our simulations, once the system reaches such minimum, further cycles do not change the state of the system and an asymprote for enthalpy is observed. Supplementary Fig. shows the stretched exponential energy drop as a function of applied cycles on the system.

To compare the results of ISM on the 1D SEL model with actual system dynamics, we run kinetic Monte Carlo simulations beginning from the same configuration as ISM runs. At each configurations, two barriers flank the system (E_{bl} and E_{br}). Following Voter[6], the movement probability is calculated by:

$$p_l(k) = \frac{e^{-E_{bl}(k)/kT}}{Z}; \qquad p_r(k) = \frac{e^{-E_{br}(k)/kT}}{Z} \tag{2}$$

where $Z = e^{-E_{bl}(k)/kT} + e^{-E_{br}(k)/kT}$ and k represents the minimum occupied. To advance simulation time after each jump, we draw a random number as follows:

$$t_k = -\frac{1}{Z}\ln(\mathrm{rand}_k) \tag{3}$$

where rand_k represents a random number drawn from a uniform distribution. This means that at each specific Monte Carlo step, the cumulative sum of previous time increments gives the elapsed simulation time. We ran 200 independent Monte Carlo simulations on the landscape and recorded enthalpy as a function of time as shown in Supplementary Fig. .

We observe a striking similarity of behavior from ISM and kinetic Monte Carlo simulations as shown in Figs. Supplementary Fig. and Supplementary Fig. . By eliminating energy between these

two functions, one can obtain a relation between cycles and time as shown in Supplementary Fig. .

We can see that when the underlying landscape has characteristics of the current simplified model, one can expect a stretched exponential behavior under ISM and kinetic Monte Carlo simulations which points to a linear time-cycle relationship. So when ISM results are at hand, one can convert them to the expected behavior under actual dynamics. We emphasize that it is not possible with a 1D model to show that the inherent structures visited during ISM are the same as those traversed by kinetic Monte Carlo simulations. As mentioned earlier, this requires a higher dimensional model (such as NK or transition matrix models[5]).

We also note that applicability of transition state theory to shear deformations of C-S-H has been mentioned by Masoero et al.[7]. However, to the best of our knowledge, we believe no rigorous proof exists as of yet. Hence, we have modified the text to address the fact that applicability of transition state theory is an assumption of our work.

Supplementary Note 3

Strain calculations. We define $\mathbf{h} = [\mathbf{a}, \mathbf{b}, \mathbf{c}]$ where the three column matrices in the braces are vectors defining the simulation cell. As the cell is strained, position of an atom changes from $\mathbf{r}_0 = \mathbf{h}_0\mathbf{s}$ to $\mathbf{r} = \mathbf{hs} = \mathbf{hh}_0^{-1}\mathbf{r}_0$ where $\mathbf{s}$ is the fractional coordinate of the atom. Hence, the displacement vector is obtained as:

$$\mathbf{u} = \mathbf{r} - \mathbf{r}_0 = (\mathbf{hh}_0^{-1} - 1)\mathbf{r}_0 \qquad (4)$$

and from the strain-displacement relation, we have:

$$\mathbf{E} = \frac{1}{2}\left[(\nabla_X \mathbf{u})^T + \nabla_X \mathbf{u} + (\nabla_X \mathbf{u})^T \cdot \nabla_X \mathbf{u}\right] \qquad (5)$$

Substituting from (4), we get:

$$\mathbf{E} = \frac{1}{2}(\mathbf{h}_0^{-T}\mathbf{G}\mathbf{h}_0^{-1} - \mathbf{I}) \qquad (6)$$

where $\mathbf{G} = \mathbf{h}^T\mathbf{h}$.

Supplementary Note 4

The Gibbs free energy is defined as $G = H - TS$ where $H = U + pV$ is the usual definition of enthalpy. However, due to the partial nanocrystallinity/quasi-glassy nature of C-S-H structure and also the fact that our simulations are carried out at room temperature, the TS contribution can be neglected. This means that the configuration at equilibrium corresponds to a minimum of

$H = U + pV$ for a system under hydrostatic pressure. Now, to be able to include all components of stress in the formulation, a generalized form on enthalpy is needed which is proposed as [13]:

$$H_{ex} = U + p(V - V_0) + V_0 tr(\boldsymbol{\sigma} - \mathbf{p})\boldsymbol{\varepsilon}$$

where $\boldsymbol{\sigma}$ is the stress tensor and V is the volume of the simulation cell. So in this minimization, cell dimensions are variable. This means that volume is not constant. If we had constant volume and neglected entropic contributions, we would be using the Helmhoz free energy, which is $A = U - TS$ and if the second term on the right is neglected, we would be minimizing the potential energy U.

Supplementary Note 5

A comparison of available models. In this section, we compare several characteristics of three of the main models currently available: M1: the model used in current study, M2: the model proposed by Kumar *et al.* [2], M3: the model proposed by Kovačević *et al.*[3].

A recent study carried out by Geng *et al.*[14] provides a summary of experimental values for basal spacing of C-S-H samples with C/S as high as 1.7.

It is observed that M1 and M2 give acceptable values but M3 shows large values. The larger values observed, we believe, is the result of using the structure of 14Å tobermorite as the starting point.

Abdolhosseini *et al.*[1] reported variations of equivalent water content (sum of hydroxyl groups and molecular water) with C/S. This allows comparison with SANS experiments experimental measurements[15]. The reactive simulations performed also allowed for calculation of molecular water. In Supplementary Fig. , we calculate both molecular and equivalent water content of the three models considered. For all models, good agreement is obtained for the case of equivalent water, however models M2 and M3 show a large discrepancy for values of molecular water.

Behavior of more crystalline C-S-H structures under ISM. The true atomic structure of C-S-H remains highly debated and as a result, several models and forcefields have been proposed to capture its macroscopic properties[10,16–19]. To study the effect of model and forcefield variation on the results of ISM, we reconstructed two of the main variants of C-S-H models apart from what we used in this paper: 1- the model proposed in Kumar *et al*[16] (M2) and 2- the model proposed by Kovačević *et al*[20] (M3). CementFF forcefield parameters[17,18] were translated into LAMMPS syntax from the original DL_POLY files included in Mishra *et al.*[18] and were used for both models.

After conversion of coordinate files and forcefield parameters to LAMMPS format, our goal is to perform the same ISM on the models and see how they behave. However, due to the large number of atoms in the simulation cell of these models, we devise an approximate version of ISM (ISM/A) to circumvent impractically long simulation times. In this method, we replace the multitude optimizations in each cycle, with a single jump to $\tau + \Delta\tau$ and a single jump to $\tau - \Delta\tau$. Supplementary Fig. show the results for the loading stage due to ISM/A. Although some level of

approximation is introduced due to use of ISM/A, the overall behavior matches what is observed in the paper for our model.

These results point to the fact that a combination of the layered structure of C-S-H and interlayer spacing determine whether we a logarithmic or viscoelastic behavior. Due to the presence of bridging sites in the more crystalline models, large sliding barriers are expected and a viscoelastic behavior seems plausible.

To further test this hypothesis and also ISM's behavior, we perform a thorough study of Hamid's 11Å Tobermorite which is defect-free. The results are shown in Supplementary Fig. and demonstrate a behavior similar to the C-S-H case with low water content.

Supplementary References

1. Abdolhosseini Qomi, M. J. *et al.* Combinatorial molecular optimization of cement hydrates. *Nat. Commun.* **5,** 4960 (2014).

2. Kumar, A. *et al.* The atomic-level structure of cementitious calcium silicate hydrate. *J. Phys. Chem. C* **121,** 17188–17196 (2017).

3. Kovačević, G., Persson, B., Nicoleau, L., Nonat, A. & Veryazov, V. Atomistic modeling of crystal structure of Ca1.67SiHx. *Cem. Concr. Res.* **67,** 197–203 (2015).

4. Perez, D., Cubuk, E. D., Waterland, A., Kaxiras, E. & Voter, A. F. Long-Time Dynamics through Parallel Trajectory Splicing. *J. Chem. Theory Comput.* **12,** 18–28 (2016).

5. Fiocco, D., Foffi, G. & Sastry, S. Memory effects in schematic models of glasses subjected to oscillatory deformation. (2015). doi:10.1088/0953-8984/27/19/194130

6. Voter, A. F. Introduction to the kinetic Monte Carlo method. in *Radiation Effects in Solids* 1–23 (Springer, Dordrecht, 2007).

7. Masoero, E. *et al.* Kinetic Simulations of Cement Creep: Mechanisms from Shear Deformations of Glasses. in *CONCREEP 10* 555–564 (American Society of Civil Engineers, 2015). doi:10.1061/9780784479346.068

8. Hamid, S. A. The crystal structure of the 11 A natural tobermorite. *Zeitschrift fur Krist. - New Cryst. Struct.* **154,** 189–198 (1981).

9. Pustovgar, E. *et al.* Understanding silicate hydration from quantitative analyses of hydrating tricalcium silicates. *Nat. Commun.* **7,** 10952 (2016).

10. Shahsavari, R., Pellenq, R. J.-M. & Ulm, F.-J. Empirical force fields for complex hydrated calcio-silicate layered materials. *Phys. Chem. Chem. Phys.* **13,** 1002–1011 (2011).

11. Richardson, I. G. The calcium silicate hydrates. *Cem. Concr. Res.* **38,** 137–158 (2008).

12. Cygan, R. T., Liang, J.-J. & Kalinichev, A. G. Molecular models of hydroxide, oxyhydroxide, and clay phases and the development of a general force field. *J. Phys. Chem. B* **108,** 1255–1266 (2004).

13. Parinnello, M. & Rahman, A. Polymorphic transitions in single crystals. *J. Appl. Phys.* **52,** 7182–7190 (1981).

14. Geng, G., Myers, R. J., Qomi, M. J. A. & Monteiro, P. J. M. Densification of the interlayer spacing governs the nanomechanical properties of calcium-silicate-hydrate. *Sci. Rep.* **7,** (2017).

15. Allen, A. J., Thomas, J. J. & Jennings, H. M. Composition and density of nanoscale calcium–silicate–hydrate in cement. *Nat. Mater.* **6,** 311–316 (2007).

16. Kumar, A. *et al.* The atomic-level structure of cementitious calcium silicate hydrate – Supporting Information Table of Contents.

17. Galmarini, S., Kunhi Mohamed, A. & Bowen, P. Atomistic simulations of silicate species interaction with portlandite surfaces. *J. Phys. Chem. C* **120,** 22407–22413 (2016).

18. Mishra, R. K. *et al.* cemff: A force field database for cementitious materials including validations, applications and opportunities. *Cement and Concrete Research* **102,** 68–89 (2017).

19. Pellenq, R. J.-M. *et al.* A realistic molecular model of cement hydrates. *Proc. Natl. Acad. Sci.* **106,** 16102–16107 (2009).

20. Kovačević, G., Nicoleau, L., Nonat, A. & Veryazov, V. Revised atomistic models of the crystal structure of C-S-H with high C/S ratio. *Zeitschrift fur Phys. Chemie* **230,** 1411–1424 (2016).
