# Supplementary material for: Nanoscale origins of creep in calcium silicate hydrates
Source: Nat Commun. 2018 May 3;9:1785. doi: 10.1038/s41467-018-04174-z (PMC5934396; doi:10.1038/s41467-018-04174-z)
Supplement: Supplementary file 2 — Description of Additional Supplementary Files [file 41467_2018_4174_MOESM2_ESM.pdf]

Description of Additional Supplementary Files

File Name: Supplementary Data 1
Description: The relaxed atomic structure of C-S-H.

File Name: Supplementary Data 2
Description: Atomic structure of C-S-H after cyclic loading through the Incremental Stress Marching technique.
